# Health-related quality of life in adults with profound postlingual hearing loss before and after cochlear implantation

**DOI:** 10.1007/s00405-021-06866-7

**Published:** 2021-06-08

**Authors:** Joanna Rostkowska, Piotr Henryk Skarzynski, Joanna Kobosko, Elzbieta Gos, Henryk Skarzynski

**Affiliations:** 1 Rehabilitation Clinic, World Hearing Center, Institute of Physiology and Pathology of Hearing, Kajetany, Warsaw, Poland; 2 Department of Special Education, The Maria Grzegorzewska University, Szczęśliwicka 40, 02-353 Warszawa, Poland; 3 Teleaudiology and Screening Department, World Hearing Center, Institute of Physiology and Pathology of Hearing, Kajetany, Warsaw, Poland; 4 Faculty of Medicine, Heart Failure and Cardiac Rehabilitation Department, Medical University of Warsaw, Warsaw, Poland; 5 Institute of Sensory Organs, Kajetany, Warsaw, Poland; 6 Oto-Rhino-Laryngology Surgery Department, World Hearing Center, Institute of Physiology and Pathology of Hearing, Kajetany, Warsaw, Poland; 7 Experimental Audiology Department, Institute of Physiology and Pathology of Hearing, Warsaw, Poland

**Keywords:** Health-related quality of life, Postlingual hearing loss, Cochlear implant, AQoL-8D questionnaire

## Abstract

**Purpose:**

In clinical trials and everyday medical practice, health-related quality of life (HRQoL) measures are increasingly being used. That is, in addition to the usual biological health assessment, the impact of disease and treatment on the patient’s functioning in the physical, mental, and social areas is an important parameter. The aim of this study was to assess HRQoL before and after using a cochlear implant (CI) in adults with profound hearing loss.

**Methods:**

There were 104 patients who qualified for the study. All gave informed and free consent. The study involved adults with bilateral hearing loss above 81 dB HL and rated according to the World Health Organization (WHO) classification scheme as having profound hearing loss (which includes deafness). In each participant, the hearing loss was postlingual, that is, it occurred after speech and language had developed. The assessment of quality of life (AQoL-8D) questionnaire was used to assess the health-related quality of life in the study participants.

**Results:**

Quality of life significantly increased (*p* < 0.001) after cochlear implantation in almost all domains (except the pain dimension). The mean increase in overall quality of life was 0.16, the greatest improvement was found in the dimensions senses (mean change of 0.17) and self-worth (mean change of 0.16).

**Conclusion:**

Cochlear implantation improves the health-related quality of life of the postlingually deaf.

## Introduction

For people with acquired deafness, performing common life tasks and engaging fully in social activity is difficult. Profound hearing loss reduces interpersonal contacts, which may result in social exclusion and related psychological consequences, e.g. depression [1–3]. To reduce the effects of profound postlingual hearing loss in adults, cochlear implants (CIs) are commonly used [4, 5]. This procedure is an effective form of medical treatment and rehabilitation [6, 7].

Most of the research on CIs in adults has focused on auditory perception before and after implantation. Results show that a CI significantly improves the identification of environmental sounds and of speech, both in silence and in noise [8–11]. However, there is also a need to gauge the subjective impact of using a CI on the psychological, social, and physical aspects of the user’s life [12–14]. Such a holistic approach to the patient can reveal an overall picture of how well they are functioning, summed up by the term ‘quality of life’ [15].

Currently, the measurement of health-related quality of life is an important way of gauging treatment efficacy and is typically used alongside medical assessment. Assessing a patient’s HRQoL means letting them express their subjective feelings. The patient’s feelings of physical and mental well-being are a good measure of the success of modern medicine. Scientifically investigating the quality of life of adults with profound postlingual hearing loss and who use a CI can provide answers as to whether (and in which areas) the device has provided a substantial change in their quality of life. The knowledge obtained can help in assessing treatment results, selecting rehabilitation and psychological methods, optimizing treatment costs, and improving the quality of medical care. Even just completing the quality of life questionnaire is of value to the patient as it prompts self-reflection about expectations and of the skills that have been acquired.

The quality of life of CI users is of interest to many researchers. Numerous scientific studies confirm an improvement in the overall quality of life of people with profound postlingual hearing loss after they acquire a CI. Such a result was reported by Lassaletta et al. [9] after using the health utilities index mark II (HUI 2) and the health utilities index mark III (HUI 3). More generally, they also noted a positive impact of cochlear implantation in other spheres of life. Lassaletta and colleagues found a significant improvement in the quality of life in the specific areas explored by HUI 3 hearing, speech, and emotions. Likewise, Orabi et al. [16] used the Glasgow health status inventory (GHSI) to find a statistically significant improvement in overall quality of life after the use of a CI. This was also noticed by Vermeire et al. [17] when using the Glasgow benefit inventory (GBI). The results obtained by them also showed a statistically significant improvement in the quality of life in the social field. They also used the hearing handicap inventory for adults (HHIA) and described improvements in emotional functioning in addition to the improved overall quality of life. Research involving the hearing participation scale (HPS) has also described a statistically significant improvement in the overall quality of life in this group of people [18]. Numerous research teams have used the Nijmegen cochlear implant questionnaire (NCIQ), Hinderink, Krabbe and Van Den Broek [19], Lassaletta, Castro, Bastarrica et al. [9], Hirschfelder, Gräbel and Olze [20], Liu, Chen, Kong, Li, Mo and Zheng [21], Looi, Mackenzie, Bird and Lawrenson [22], and Sanchez–Cuadrado, Gavilan, Perez–Mora et al. [23]. All agree that after a CI, the quality of life of the patients improves significantly in the physical, psychological, and social domains (as described by the questionnaire’s authors) [9, 19–23]. Hogan et al. [24] also found an improvement in the quality of life but noted that it did remain lower than the population norm.

In our research on the health-related quality of life of CI users with profound postlingual hearing loss, we decided to use the assessment quality of life questionnaire with its eight dimensions (AQoL-8D) [25] for several reasons. Among the tools used so far to measure the health-related quality of life from a multi-attribute utility instrument (MUI) group, AQoL-8D covers the broadest range of psychological and social functioning [25–27]. AQoL-8D is one of the newest tools in this group and enables a utility index to be calculated. This allows one to compare HRQoL results with those from other groups who have chronic illness or disability, as well as with the results from related research that has used other multi-attribute utility instruments (but not AQoL-8D). For example, there have been qualitative studies involving people with spinal cord injury, which have been studied using various preference-based quality of life instruments, and these have indicated that AQoL-8D reveals a generally positive perception in this group of people, i.e. it is “comprehensive without being burdensome” [28]. This ability is also a significant advantage in undertaking research on people with profound postlingual hearing loss. Finally, there is a Polish adaptation of AQoL-8D and standards for otolaryngological patients have recently been published [29].

The aim of this study was to assess the health-related quality of life of adults with profound hearing loss (acquired after mastering language) before and after receiving a CI.

## Methods

For this study, we enrolled and qualified 104 patients (58 women and 46 men) who attended the Institute of Physiology and Pathology of Hearing. All gave their informed and free consent. The study involved adults with bilateral hearing loss above 81 dB HL who had profound hearing loss (or deafness) according to the World Health Organization (WHO) classification [30]. In each participant, the hearing loss began after speech and language had developed (i.e., postlingually). Their ages ranged from 6 to 69 years of age (M = 34.8, SD = 16.6). Each person was qualified for treatment with a CI. After the CI surgery, each patient in the study group declared systematic use of the device. The youngest person in the group was 28 years old at the time of implantation and the oldest was 70 (M = 54.4, SD = 12.4). The time of CI use was between 0.7 and 3 years (M = 1.8, SD = 0.6). The duration of profound hearing loss ranged from 2 to 53 years (M = 21.2, SD = 14.2).

### Assessment of quality of life (AQoL-8D)

The AQoL-8D was used to assess the health-related quality of life in adults using a CI. AQoL-8D was created by Hawthorne and colleagues to improve the sensitivity of psycho-social health [18] and is a comprehensive preference-based measure of HRQoL. It contains eight dimensions and can define a total of 2.4 × 1023 health states. AQoL-8D has been adapted to the polish language [29].

With AQoL-8D, it is possible to assess the overall quality of life as well as the quality of life in the following dimensions: independent living, pain, senses, mental health, Happiness, coping, relationships, and self-worth. The dimensions can be grouped into two superdimensions: physical (independent living, pain, senses) and psychosocial (mental health, happiness, coping, relationships, self-worth). AQoL-8D consists of 35 items. The respondents select a statement best describing their situation in the last week from a range of 4–6 options depending on the number of items. AQoL-8D was scored based on an Australian-devised scheme [31]. The questionnaire enables the determination of a utility index (UI) ranging from 0 to 1, meaning that an economic analysis of the medical technology under study can be made. The authors of AQoL-8D provide an example of assigning a utility weight to item five of the questionnaire: how often do you feel sad? An answer ‘never’ has the utility weight of one, ‘rarely’ −0.86, ‘sometimes’ −0.58, ‘often’ −0.20, while an answer ‘nearly all the time’ has zero utility weight [32]. AQoL-8D is recommended by the agency for health technology assessment, according to which clinical trials on the effectiveness of new medical technologies must also include an assessment of the improvement in quality of life [29]. AQoL-8D has been used to assess the quality of life in adults using bilateral active middle ear implants, people with non-implantable, pressure-free, adhesive bone conduction hearing aids, and older adult patients (≥ 65 years) who use hearing aids [33–35]. The questionnaire can also be used to study the health-related quality of life of people with chronic diseases such as diabetes, obesity, depression, and cancer [36–38].

Patients completed the AQoL-8D questionnaire twice. The first was during a diagnostic hospitalization qualifying them for treatment with a CI. The second time was 8 months to 3 years after the speech processor was connected.

### Statistical analysis

Descriptive statistics were calculated for the analyzed variables. They were minimum (Min) and maximum (Max), mean (M), median (Me), standard deviation (SD), skewness (SK), and kurtosis (K). The normality assumption was checked with the Kolmogorov–Smirnov test. To compare the quality of life before and after cochlear implantation a parametric *t* test for paired samples was used (when the normality assumption was met) or a nonparametric Wilcoxon signed-rank test was applied (if a variable did not fit a normal distribution). The change in the quality of life was calculated by subtracting the initial preoperative score from the follow-up postoperative score, a positive result indicated improvement and a negative result of deterioration in the quality of life. A *p* value below 0.05 was considered statistically significant. Statistical analysis was performed using IBM SPSS Statistics (V. 24.0).

## Results

Table 1 presents data on the comparison of the health-related quality of life measured with the AQoL-8D questionnaire before and after CI surgery.

**Table 1:** Comparison of the quality of life measured with the AQoL-8D questionnaire before and after cochlear implantation

Dimension	Before cochlear implantation				After cochlear implantation				Test result
	Min	Max	M	SD	Min	Max	M	SD	
Independent living	0.39	1.00	0.78	0.15	0.35	1.00	0.86	0.14	5.75***
Pain	0.15	1.00	0.76	0.24	0.21	1.00	0.78	0.22	0.82
Senses	0.25	0.97	0.53	0.18	0.35	0.97	0.71	0.16	6.92***
Mental health	0.25	1.00	0.53	0.16	0.29	1.00	0.60	0.13	4.67***
Happiness	0.24	1.00	0.64	0.18	0.36	1.00	0.73	0.13	5.37***
Coping	0.38	1.00	0.72	0.17	0.52	1.00	0.80	0.12	4.80***
Relationships	0.47	1.00	0.60	0.14	0.47	1.00	0.70	0.14	6.44***
Self-worth	0.28	1.00	0.64	0.21	0.39	1.00	0.81	0.14	6.88***
Physical superdimension	0.15	0.91	0.50	0.20	0.18	0.96	0.62	0.20	6.00***
Psycho-social superdimension	0.05	0.92	0.27	0.19	0.09	1.00	0.37	0.18	5.73***
Overall quality of life	0.17	0.99	0.51	0.23	0.21	0.99	0.66	0.19	6.44***

*Min* minimum score, *Max* maximum score, *M* mean, *SD* standard deviation

***p < 0.001

The overall quality of life (UI) was on average 0.51 before cochlear implantation, with a standard deviation of about 45% of the mean. The patients’ functioning was better in the physical domain (mean 0.50) than in the psycho-social domain (mean 0.27). The subjects achieved the highest results in the dimensions of Independent living, pain, and coping, the lowest scores were in the dimensions senses and mental health*.* The greatest variation (expressed as the ratio of standard deviation to the mean) was noted in the psycho-social superdimension.

After cochlear implantation, the overall quality of life (UI) was on average 0.66, with quite small variation (standard deviation was 29% of the mean). The subjects achieved the highest results in the dimensions of independent living, self-worth, and coping and the lowest in the dimension mental health.

Statistically significant differences were found in almost all dimensions of health-related quality of life (except pain). Significant improvement (*p* < 0.001) was observed in the dimensions of independent living, sense, mental health, happiness, coping, relationships, and self-worth, and also in both superdimensions. The overall quality of life (UI) significantly increased after cochlear implantation.

Table 2 presents data on the size of changes in the quality of life measured with AQoL-8D after CI surgery.

**Table 2:** Descriptive statistics on the size of changes in the quality of life measured with the AQoL-8D questionnaire after cochlear implantation

Changes in dimensions	Min	Max	M	SD	Me	SK	K
Independent living	− 0.28	0.47	0.08	0.13	0.07	− 0.05	0.62
Pain	− 0.41	0.85	0.03	0.20	0.00	0.91	2.14
Senses	− 0.54	0.54	0.17	0.19	0.20	− 0.85	1.47
Mental health	− 0.31	0.36	0.07	0.14	0.08	− 0.47	0.21
Happiness	− 0.31	0.42	0.10	0.16	0.07	− 0.01	− 0.07
Coping	− 0.26	0.51	0.08	0.15	0.04	0.63	0.41
Relationships	− 0.24	0.40	0.10	0.13	0.10	0.05	− 0.36
Self-worth	− 0.33	0.54	0.16	0.19	0.16	− 0.02	− 0.32
Physical superdimension	− 0.41	0.61	0.13	0.19	0.11	− 0.13	0.45
Psycho-social superdimension	− 0.35	0.49	0.11	0.17	0.12	− 0.51	0.54
Overall quality of life	− 0.43	0.62	0.16	0.20	0.16	− 0.42	0.49

*Min* minimum score, *Max* maximum score, *M* mean, *SD* standard deviation, *Me* median, *SK* skewness, *K* kurtosis

The change in overall quality ranged from –0.43 to 0.63, the average was 0.16, although the dispersion was large. The greatest change in quality of life was found in the senses and self-worth dimensions. In contrast, the smallest change was observed in the pain dimension.

## Discussion

The aim of the study was to assess the health-related quality of life before and after cochlear implantation applying a multi-attribute utility instrument, i.e. AQoL-8D [31], on adults with profound hearing loss acquired after mastering language. To the best of our knowledge this is the first study using AQoL-8D on a population of postlingually deaf CI users.

Our findings were that the overall quality of life in the studied group after cochlear implantation improved significantly compared to measurements before implantation. The level of overall quality of life (utility index, UI) for the group of respondents before implantation was similar to results obtained with AQoL-8D on participants with depression [26, 27]. It is known that people with profound postlingual hearing loss (mostly without a CI) suffer from significantly higher levels of depressive symptoms compared to the general population [39, 40]. However, adults with profound postlingual hearing loss who use a CI show a degree of psychological distress on a level with that of the general population, including depression, as demonstrated by other Polish studies on postlingually deaf CI users [3] and related work [41].

The utility index as measured by AQoL-8D, obtained in our study, amounted to an average of 0.66 after implantation, similar to values obtained with the same tool on people with chronic illnesses such as asthma, athritis, cancer [26], diabetes [26, 42], heart disease [26, 43], and patients after bariatric surgery [44]. Health-related quality of life (AQoL-8D) of the profoundly deaf CI users from our study does not differ appreciably from the quality of life of other otolaryngological patients studied in Poland [29].

However, compared to healthy populations from a large study of health and subjective wellbeing conducted across four countries (UK, USA, Canada, Australia) [26], the quality of life in the postlingually deaf after a CI was significantly lower (*t *test, *p* < 0.001). A similar result is expected for the healthy Polish population, although caution must be applied as there are no Polish standards for AQoL-8D so far, and socioeconomic and cultural factors that determine the level of QoL in a nation’s population will also be important.

The quality of life in the studied group of postlingually deaf CI users significantly improved in the areas of independent living, senses, mental health, happiness, self-worth, coping, and relationships, and in the two superdimensions of AQoL-8D (physical and psycho-social). The psycho-social QoL was much lower than in the physical QoL, showing that in people with profound postlingual hearing loss, difficulties in this area are particularly important [2, 3, 39, 40, 45]. The biggest changes in the quality of life after receiving a CI were noted in the dimensions of self-worth, senses, and relationships. The relationship between the quality of life and self-worth is widely described in the literature—the quality of life increases with higher self-worth, which is the case among people with motor disabilities [46] and adolescents with chronic disease [47]. It should be mentioned that self-esteem (as assessed by the Rosenberg self-esteem scale, RSES) has been shown to remain significantly lower in profoundly deaf CI users than in the general population [3, 45], even though our findings with AQoL-8D indicated a significant improvement after a CI—there was a positive change in the dimension of self-worth. However, it is worth noting that self-worth (AQoL-8D) and self-esteem (RSES) differ slightly—self-worth includes the aspect of “being a burden”, and after a CI this feeling can be reduced. This aspect could be investigated empirically in the future.

All our results using AQoL-8D clearly indicate a significant improvement in quality of life after implantation in the vast majority of distinct dimensions. The exception was the dimension of Pain, which remains at a similar level—probably because physical pain does not accompany postlingual deafness, unlike pain associated with other diseases. Other studies, which have used various questionnaires to measure the quality of life, confirm improvements in the overall quality of life of CI patients who previously had profound postlingual hearing loss [16–18].

## Conclusions

Cochlear implantation improves the quality of life, which means that for the postlingually deaf, it is the appropriate method of treatment. However, adults with acquired profound postlingual hearing loss who use a CI might still require psychological intervention to improve their quality of life, especially in the area of psychosocial functioning. In the future, further research on this population should take into account socio-demographic factors: age, gender, education, marital and partnership status, employment status, and cultural and psychosocial factors. These factors can be significant for the health-related quality of life experienced by people with chronic illness or disability, and that includes postlingually deaf CI users.

## Data Availability

The data that support the findings of this study are available from the corresponding author (JR) upon request.
